# Successful Removal of the Uterus by Ligature

**Published:** 1844-10-05

**Authors:** 


### SUCCESSFUL REMOVAL OF THE UTERUS BY LIGATURE.

Dr. Toogood, of Bridgewater, narrates in the Provincial Medical Journal, the case of a maiden lady, sixty years of age, whom he had attended several years previously for prolapsus uteri. A former partner of his, Mr. Parsons, was called to her in consequence of procidentia uteri occurring, and her being totally unable to restore the womb to its proper position, in which he also failing, a consultation was held with Dr. Toogood, and it was determined to remove the protruded mass by ligature, which was applied very firmly round the neck of the swelling, just within the vagina. The mass, when removed, was about two pounds' weight, and of the shape of the uterus, but its structure was much altered in character, the cavity being quite obliterated, and the os uteri almost cartilaginous. The patient perfectly recovered.

